# High-throughput transformation pipeline for a Brazilian *japonica* rice with bar gene selection

**DOI:** 10.1007/s00709-014-0741-x

**Published:** 2014-12-07

**Authors:** B. Dedicova, C. Bermudez, M. Prias, E. Zuniga, C. Brondani

**Affiliations:** 1 International Center for Tropical Agriculture A.A. 6713, Cali, Colombia; 2 Embrapa Arroz e Feijão, Rodovia GO-462, Km 12 75.375-00, Santo Antônio de Goiás, Goiás Brazil

**Keywords:** *Oryza sativa* L, Upland rice, *Agrobacterium*-mediated transformation, Bar selection, Backbone integration

## Abstract

The goal of this work was to establish a transformation pipeline for upland Curinga rice (*Oryza sativa L. ssp*. japonica) with bar gene selection employing bialaphos and phosphinothricin as selection agents. The following genes of interest: *AtNCED3*, *Lsi1*, *GLU2*, *LEW2*, *PLD*-*alpha*, *DA1*, *TOR*, *AVP1*, and *Rubisco* were cloned into the binary vector p7i2x-Ubi and were transferred into *Agrobacterium* strain EHA 105. Embryogenic calli derived from the mature embryos were transformed, and transgenic cells and shoots were selected on the medium supplemented with bialaphos or phosphinothricin (PPT) using a stepwise selection scheme. Molecular analyses were established using polymerase chain reaction and Southern blot for the bar gene and the NOS terminator. Overall, 273 putative transgenic plants were analyzed by Southern blot with 134 events identified. In total, 77 events had a single copy of the transgene integrated in the plant genome while 29 events had two copies. We tested backbone integration in 101 transgenic plants from all constructs and found 60 transgenic plants having no additional sequence integrated in the plant genome. The bar gene activity was evaluated by the chlorophenol red test and the leaf painting test using phosphinothricin with several transgenic plants. The majority of T0 plants carrying the single copy of transgene produced T1 seeds in the screen house.

## Introduction

Rice equates to life for thousands of millions people in Asia alone where more than 2000 million people obtain 60 to 70 % of their calories from rice and its products (FAO 2013). In Latin America, rice is the most important food grain providing 27 % of daily calorie intake overall, ranging from 8 % in Central America to 47 % in the Caribbean region (FAOSTAT 2012). Upland rice, known as aerobic rice, is grown in South America, Africa, and Asia (Fageria 2002). In the central part of Brazil (Cerrados), upland rice plays an important role in the cropping system whereby it is first grown after clearing land for pasture (Fageria 2010). The main limiting factors for adopting high-yielding rice varieties is drought and access to nitrogen in drought-prone rainfed rice environments (Fageria 2009). These two traits, drought tolerance and nitrogen-use efficiency, have been of high interest in past experimental research (Campbell et al. 1995; Umezawa et al. 2006; Serraj et al. 2008) and have been reviewed several times in recent years (Hadiarto and Tran L-S 2011; Lawlor 2013). Dozens of genes with different functions and modes of action have been identified (Shinozaki and Yamaguchi-Shinozaki 2007; Yang et al. 2010; Jeong et al. 2013) with several going through confined field testing (Deikman et al. 2011; Gaudin et al. 2013).

This study focused on following genes linked to plant stress resistance, plant growth, and yield: The *AtNCED3* encodes the key enzyme in the abscisic acid (ABA) biosynthesis via overexpression of 9-cis-epoxycarotenoid dioxygenase in Arabidopsis (Iuchi et al. 2001). The *Lsi1* gene (Low silicon rice 1) encodes the Si transporter from the aquaporin gene family, is expressed in rice roots (Ma et al. 2006), and is downregulated during dehydration stress by ABA (Yamaji and Ma 2007). The *AtCEsA8*/*IRX1* gene from the Lew2 Arabidopsis mutants (Chen et al. 2005) is one of ten genes essential for the cellulose synthase complex in the secondary cell walls (Taylor 2008). The phospholipase *Dα1* (*PLD α1*) gene is involved in the stress response through stomata closure directed by the ABA effect (Mishra et al. 2006; Uraji et al. 2012). Expression of the *AtTOR* kinase (target of rapamycin) influences seed and plant growth and controls resistance to osmotic stress (Deprost et al. 2007). Disruption of the *TOR* activity can lead to premature arrest of endosperm and embryo development (Menand et al. 2002). Another gene controlling seed and organ size is the *DA1* gene, which encodes the ubiquitin receptor that controls the cell proliferation period (Li et al. 2008) and final seed and organ size as well as increases plant biomass. *AVP1* encodes a vacuolar pyrophosphatase which functions as a proton pump in the vacuolar membrane and, in transgenic Arabidopsis plants, expression of this gene can increase the vacuolar proton gradient resulting in elevated solute accumulation and water retention (Gaxiola et al. 2001). If this gene is overexpressed in cotton, transgenic plants display improved tolerance to drought and salt stress (Pasapula et al. 2011). The major role of the *AtGLU2* gene is nitrogen assimilation in plant roots (Coschigano et al. 1998; Lancien et al. 2002). A positive correlation between nitrogen content and photosynthetic capacity through RuBisCO (ribulose-1, 5-bisphosphate carboxylase/oxygenase) is well-documented in higher plants (Evans 1989). In rice, the multiple gene family *rbcS* consists of five genes whose expression is enhanced by increased nitrogen (Suzuki et al. 2007; Miyazaki et al. 2013). The relationship between the *rbcS* and *rbcL* genes leads to leaf senescence and nitrogen influx (Imai et al. 2008).

The bar gene-based selection system produced numerous herbicide-resistant biotech crops, e.g., oilseed rape (De Block et al. 1989; Kopertekh et al. 2009), lettuce (McCabe et al. 1999), soybean (Zeng et al. 2004), mungbean (Sonia et al. 2007), carrots (Jayaraj et al. 2008), sweet potato (Zang et al. 2009), sugarcane (Joyce et al. 2010), cassava (Koehorst-van Putten et al. 2012), and tomato (Khuong et al. 2013).

The bar gene selection was also successfully used for conifers, *Pinus radiate* (Charity et al. 2005), orchids (Knapp et al. 2000), and flower species (Kamo and Young 2009; Chen et al. 2010b).

Among the monocotyledonous plants, e.g., wheat (Weeks et al. 1993; Wu et al. 2008), maize (Zhang et al. 1996), barley (Wu et al. (1998), oat (Kuai et al. 2001), Bermuda grass (Hu et al. 2005), tropical maize (Valdez-Ortiz et al. 2007), and ryegrass (Patel et al. 2013) were reported. The rice transformation protocols using *Agrobacterium tumefaciens* technology were widely established for several genotypes with either hygromycin (Hiei et al. 1994) or phosphinothricin (PPT) (Toki et al. 1992) selection and continue to represent two dominate selection systems currently used for this crop (Bajaj and Mohanty 2005; Chen et al. 2010a; Twyman et al. 2002).

In this study, we used ten different genes of interest as described above, which are involved in abiotic stresses mainly drought tolerance, including the ABA and nitrogen signaling pathway and plant growth. Our goal was to establish an *Agrobacterium*-mediated transformation protocol for Curinga, a commercial upland rice variety from Brazil in combination with bar gene selection using a PPT and bialaphos stepwise selection scheme.

## Materials and methods

### Plant material

The donor Curinga plants for the mature embryo/seed production were grown in the screen house at temperature 32–35 °C/20 °C (day/night) with a 12/12 photoperiod. Surface sterilized mature embryos were cultured scutellum side up for 2 weeks on Chu (Chu 1978) callus induction medium (Table 1). After 15 days, induced embryogenic calli were subcultured to the same medium and cultures proliferated for another 15 days in the same culture conditions at 24–26 °C in darkness. Well-developed embryogenic calli were subcultured 3 days prior to the transformation experiments to the same Chu callus induction medium supplemented additionally with 100 μM acetosyringone and were maintained in darkness at 24–26 °C. For composition of all media used in this study, see Table 1.

**Table 1:** Media used

Medium	Abbreviation	Composition
Callus induction	Chu-Ind.	Chu medium (macro, micro elements, vitamins), 500 mg/l l-proline, 500 mg/l l-glutamine,300 mg/l casein enzymatic hydrolyzate, 100 mg/l myo-inositol, 30 g/l maltose, 2.5 mg/l 2,4-D, 3 g/l gelrite, pH 5.8
Pre-culture	Chu + AS	as Chu-Ind. plus 100 μM acetosyringone, pH 5.8
YEP		5 g/l yeast extract,10 g/l peptone, 5 g/l NaCl,15 g/l bacteriological agar, pH 7
Chu-Infection	Chu-Inf.	Chu medium (macro, micro elements, vitamins), 2 mg/l 2, 4-D,1 g/l casamino acids, 15 g/l maltose, 15 g/l glucose, pH 5.2
Co-culture	Chu + AS	as Chu-Ind. plus 100 μM acetosyringone, pH 5.8
Washing	Chu-W	Chu medium (macro, micro elements, vitamins, 500 mg/l cefotaxime, pH 5.8
Selection	Chu S1, S2	Chu-Ind. plus3, 5 mg/l PPT or bialaphos, 250 mg/l cefotaxime, pH 5.8
Shoots induction	MS Ind.	MS medium (macro, micro elements, vitamins), 1 mg/l NAA 4 mg/l kinetin, 250 mg /l cefotaxime, 3 mg/l PPT or bialaphos, pH 5.8,
Shoots rooting	MS-R	MS medium (macro, micro elements, vitamins) hormone free, 250 mg/l cefotaxime, 5 mg/l PPT or bialaphos, pH 5.8

### PPT and bialaphos tissue sensitivity test

Different concentrations of PPT or bialaphos in Chu medium and MS medium (Murashige and Skoog 1962) were tested for different stages of rice culture development: embryogenic calli proliferation on Chu medium supplemented with 3, 5, and 7 mg/l and MS medium for the shoots’ induction, proliferation, and rooting supplemented with 1, 3 and, 5 mg/l. The lethal dose (LD) 50 and 100 were determined for different developmental stages, and those concentrations were used in a stepwise selection scheme for transformation experiments to select transgenic cells and induced shoots allowing them to form roots in the presence of the selection agent.

### Constructs, transformation, and plant selection

**Fig. 1:**
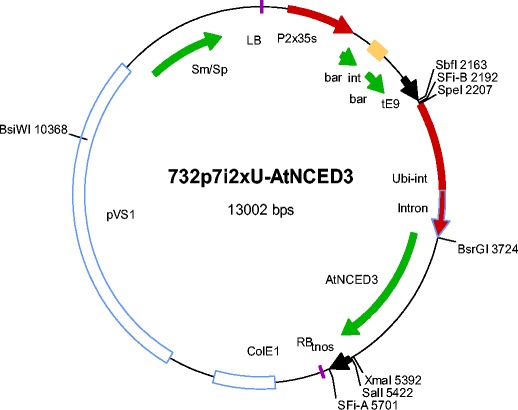
Schematic representation of the binary vector with *AtNCED*3 gene

The binary vector p7i2xU was used in combination with the following genes of interest: *AtNCED3*, At*GLU2*, *Lsi1*, *LEW2*, *PLD*-*alpha*, *DA1*, *TOR*, *AVP1*, *and* two *Rubisco* genes (provided by Dr. Hermann Schmidt from DNA Cloning Service, Germany) were driven by maize Ubi-1 promoter and selection marker gene. The bar gene was driven by doubled CaMV 35 S promoter in all constructs (Fig. 1). Ten constructs were individually transferred into *A. tumefaciens* strain EHA 105 by electroporation (Wen-jun and Forde 1989).

*A. tumefaciens* strain EHA 105 carrying binary vector was cultured on minimum AB medium (Chilton et al. 1974) supplemented with the following antibiotics: 100 μg/ml spectinomycin, 300 μg/ml streptomycin, and 60 μg/ml rifampicin. Plates were cultured at 28 °C until individual colonies were visible. One colony was used to prepare a yeast extract-peptone (YEP) plate which was subsequently used to prepare YEP liquid culture 24 h before the transformation experiments. Next morning, *Agrobacterium* cultures were centrifuged (10 min 4000 rpm at room temperature), and pellets were resuspended with liquid filter-sterilized Chu infection medium supplemented with 15 g/l maltose and glucose, 1 g/l casamino acid, and 100 μM acetosyringone (pH 5.2). *VirG* genes were induced at 21 °C between 1.5 and 3 h. Individual embryogenic rice calli were inoculated with approximately 20 μl *Agrobacterium* suspension, O.D. 0.5 (at room temperature) and after 45–50 min access, *Agrobacterium* suspension was blotted dry with sterile filter paper.

Cocultivation was carried out in darkness at 21 °C for 3 days. The infected embryogenic calli after washing with liquid Chu medium contain cefotaxime were transferred directly to Chu selection media supplemented with 3 mg/l PPT or bialaphos following by transfer to the fresh medium with 5 mg/l PPT or bialaphos after 15 days. All callus cultures were maintained in darkness at 24–26 °C.

Well-growing calli in the presence of the selection agent were transferred to MS shoots induction medium containing 3 mg/l PPT or bialaphos supplemented with 1 mg/l naphthalene acetic acid (NAA) and 4 mg/l kinetin. Cultures were gradually moved from darkness into full light conditions with 12/12 h photoperiod and light intensity 1450 μmol m^−2^ s^−1^. Green shoots were rooted in the presence of 5 mg/l PPT or bialaphos on MS growth hormone free medium.

## Molecular analyses

### DNA extraction

Following growth under hydroponic conditions for 1 week, young leaves were collected for DNA extraction. The extraction was done according to the rice DNA extraction protocol by Dr. Mathias Lorieux, International Center for Tropical Agriculture (CIAT) (personal communication). Accordingly, 450 μl of extraction buffer (100 mM Tris-HCl, pH 8.0; 1.4 mM NaCl; 20 mM EDTA, pH 8.0; 1 % polyethylene glycol, wt 8000; 2 % alkyltrimethylammonium bromide, ≥ 95 %; 0.5 % sodium hydrogensulfite) were added to each tube containing 150 mg of ground leaf tissue and incubated at 74 °C for 30 min. Then, 480 μl of chloroform/isoamyl alcohol (24:1) was added and mixed for 5 min. After centrifugation at room temperature for 30 min (4000 rpm), the supernatant was transferred to a new tube and precipitated by using 270 μl isopropanol for 1 h at 20 °C. Samples were centrifuged at 10 °C for 30 min (4000 rpm), and the pellet was washed with 70 % ethanol and resuspended in nuclease free water. Re-precipitation with ammonium acetate provided good quality DNA suitable for Southern blot analysis.

### Polymerase chain reaction analyses

Amplification of the bar gene region by conventional polymerase chain reaction (PCR) was done as an initial screening to select potential transgenic events with the following primers:

$$ \begin{array}{c}\hfill \mathrm{Bar}\hbox{-} \mathrm{F}\mathrm{w}\hbox{-} 3:\mathbf{5}^{\prime}\hbox{-}\ \mathrm{GCACGCAACGCCTACGACTGG}\hbox{-} \mathbf{3}^{\prime}\hfill \\ {}\hfill \mathrm{Bar}\hbox{-} \mathrm{R}\mathrm{v}\hbox{-} 3:\mathbf{5}^{\prime}\hbox{-}\ \mathrm{TCAGATCTCGGTGACGGGCAG}\hbox{-} \mathbf{3}^{\prime}\hfill \end{array} $$

PCR conditions were performed with settings of initial denaturation at 95 °C for 2 min, 35 cycles each at 94 °C for 30 s, 58 °C for 30 s, and 72 °C for 30 s with final extension at 72 °C for 5 min. PCR products were checked in 1 % agarose gel.

### Southern blot analyses

In order to establish copy number of bar gene and to identify independent transgenic events, 10 μg DNA of each PCR-positive plant was digested using either EcoRI or SmaI (5U/ug DNA). Because these enzymes only cut once into T-DNA without cutting the bar gene, they can be used to determine the copy number of bar gene. Digested DNA was separated in a 0.8 % agarose gel and blotted on a positively charged membrane nylon (Hybond-N+, Nylon Membranes, Positively Charged, *GE Healthcare Bio*-*Sciences Corp*). Membranes were hybridized with digoxygenin-labeled PCR probe for bar overnight and then incubated with the alkaline phosphate conjugated anti-digoxygenin antibody. After washing, blocking, and chemiluminescent reaction, the membranes were exposed to X-ray film for a minimum of 4 h.

Synthesis and labeling of probe was done following the instructions specified by the kit (PCR DIG Probe Synthesis Kit instructions, Roche). Primer bar used in PCR was also used to synthesize the bar DIG probe.

### Integration of backbone sequences

PCR amplification of backbone regions linked to T-DNA was performed. For each border, three backbone regions were amplified ranging from the nearest region to the border to almost 1000 pb from border (Table 2). PCR conditions were performed with settings of initial denaturation at 95 °C for 2 min, 35 cycles at 94 °C for 30 s, annealing temperature (specific to each set of primers) for 30 s, and 72 °C for 30 sec or 1 min. Final extension was at 72 °C for 5 min. PCR products were checked in 1.2 % agarose gel.

**Table 2:** Amplification of backbone regions

Primer	Sequence (5′-3′)	Distance (bp) outside RB
RB 17 pb Rv	ACGCTCTTTTCTCTTAG	17
T-DNA 141 bp Fw	TAGCGCGCAAACTAGGATAAA	
RB 101 pb Rv	GAACCCTGTGGTTGGCAT	101
T-DNA 141 bp Fw	TAGCGCGCAAACTAGGATAAA	
RB 867 bp Rv	GATTAGCAGAGCGAGGTATGTAG	867
T-DNA 112 bp Fw	CGCGGTGTCATCTATGTTACTA	
Primer	Sequence (5′-3′)	Distance (bp) outside LB
LB 20 pb Fw	GGGTGCAAAGCGGCAGCGGC	20
T-DNA 120 bp Rv	ATAACGCTGCGGACATCTAC	
LB 97 pb Fw	ACATGGCTCAGTTCTCAATGG	97
T-DNA 50 bp Rv	TGACCATCATACTCATTGCTGAT	
T-DNA 120 bp Rv	ATAACGCTGCGGACATCTAC	935
LB 935 bp Fw	GATCGACATTGATCTGGCTATCT	

### Chlorophenol red test

A chlorophenol red assay was used to verify the bar gene activity in selected putative transgenic plants (Wright et al. 2001) and untransformed controls. Leaf segments were placed into individual wells of a multi-well plate containing half concentration of MS medium supplemented with 25 mg/l chlorophenol red, 3 mg/l bialaphos, and 8 g/l bacto agar, pH 6.2 and were incubated at 26 °C for 24 h.

### PPT leaf painting test

The T0 plants and untransformed controls grown in the screen house were tested with 2 % aqueous solution of PPT containing 0.1 % (*v*/*v*) Tween-20 (Rasco-Gaunt et al. 1999) as well with aqueous solution of 0.1 % Tween-20. Leaf tissue response to PPT presence was scored after 1 week.

## Results

### Effect of PPT and bialaphos on rice tissues

**Fig. 2:**
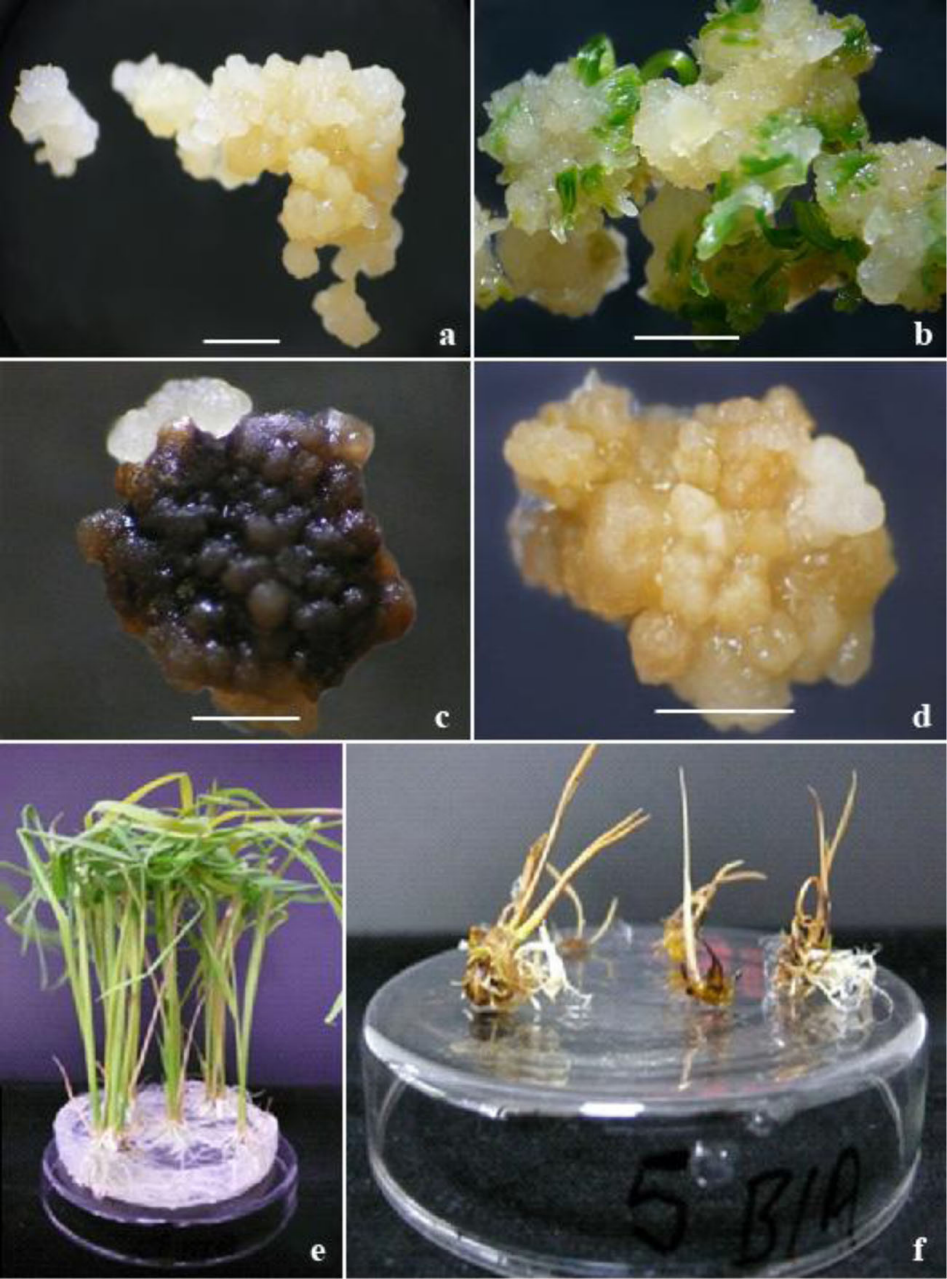
Curinga tissues’ sensitivity test. **a** Callus induction on Chu medium, control. **b** Shoots induction on MS medium, control. **c** Callus induction with 3 mg/l bialaphos. **d** Shoots induction with 3 mg/l bialaphos. **e** Rooting on MS medium, control. **f** Rooting with 5 mg/l bialaphos (*scale bar* in mm)

Prior to the transformation experiments, we tested untransformed Curinga tissue sensitivity to both selective agents, PPT and bialaphos, in Chu medium for callus proliferation and MS medium for shoots induction and rooting (Fig. 2). Parallel control cultures were carried out on media without selective agents (Fig. 2a–c). Three concentrations of PPT and bialaphos (1, 3, and 5 mg/l) were tested on callus proliferation in the Chu medium. The callus cultures were transferred to the fresh medium with increased concentration of PPT or bialaphos every 15 days. A strong tissue necrosis became visible after the second transfer (Fig. 2d). The shoots induction was originally tested with 10 and 20 mg/l PPT or bialaphos on MS medium. These concentrations were too high for Curinga calli so no shoots formed (Fig. 2e). Consequently, the LD 100 was determined for PPT and bialaphos as 3 mg/l for shoots induction and 5 mg/l for roots formation (Fig. 2f).

### Transgenic plant selection

A minimum of four transformation experiments were performed with ten constructs where embryogenic calli from approximately 50 mature embryos were induced and subcultured. We selected 302 putative transgenic plants with either mixed selection scheme where both PPT and bialaphos were used or where only one selection agent was used through the entire selection process. Callus selection was done on Chu medium supplemented with 3 mg/l PPT or bialaphos. Fifteen days following selection, pressure was increased to 5 mg/l in the second subculture. We also performed experiments with two calli subcultures with 3 mg/l PPT, followed by a third one with 5 mg/l PPT in 15-day intervals.

When we statistically analyzed (chi-square test using SAS version 9.2 on the Linux platform) the number of transgenic plants obtained from these two different selection schemes, the results showed no statistically significant difference between the experiments. Furthermore, there was no statistically significant difference between the experiments carried out with PPT, bialaphos, or with the mix of both selection agents during the entire selection process (data not shown).

**Fig. 3:**
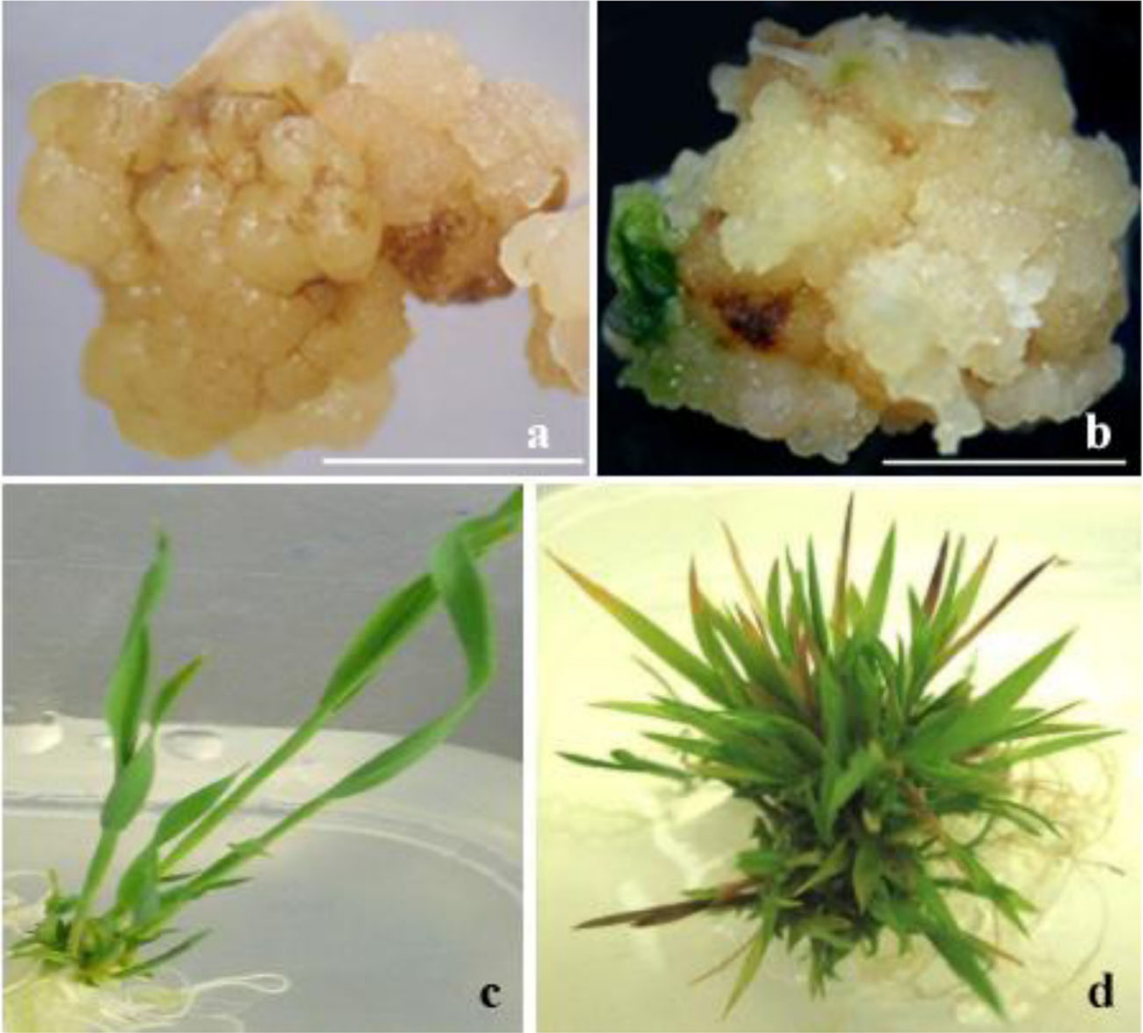
Curinga transgenic tissues. **a** p7i2xU-*Lew*2gene callus proliferation with 3 mg/l bialaphos. **b** p7i2xU-*Rubisco 1* gene shoots induction with 3 mg/l bialaphos. **c** p7i2xU-*GLU*2 gene shoots elongation with 3 mg/l bialaphos. **d** p7i2xU-*AtNCED*3 gene rooting with 5 mg/l bialaphos (*scale bar* in mm)

When we analyzed the number of calli needed to produce one transgenic plant depending on the construct and selection agent, the results showed significant differences (Table 3). The PPT selection worked better with the following constructs p7i2xU-*AVP*1, *At*NCED3, p7i2xU-DA1, and p7i2xU-TOR in difference bialaphos was better for *AtGLU*2, p7i2xU-*Lsi1*, p7i2xU-*PLD*-*alpha*, and both p7i2xU-*Rubisco* genes. The mixed selection worked similar to the PPT only for p7i2xU-*AVP*1 construct. For the shoots induction, we used MS medium supplemented with 3 mg/l PPT or bialaphos and for rooting the concentration of selection agents was increased to 5 mg/l (Fig. 3a–d).

**Table 3:** Number of calli needed to obtain one transgenic plants when PPT (PPT), bialaphos (BIA), or mixture of both selection agents (PPT-BIA) have been used

Selection agent	Construct	*F* ^a^	Number of calli
BIA	p7i2xU-*AVP*1	0.01603	186
PPT	p7i2xU-*AVP*1	0.03475	85
PPT-BIA	p7i2xU-*AVP*1	0.03333	89
BIA	p7i2xU-*At*GLU2	0.00493	607
PPT	p7i2xU-*At*GLU2	0.00402	745
BIA	p7i2xU-*At*NCED3	0.01542	193
PPT	p7i2xU-*At*NCED3	0.02370	125
PPT-BIA	p7i2xU-*At*NCED3	0.00164	1829
BIA	p7i2xU-*DA*1	0.00503	595
PPT	p7i2xU-*DA*1	0.03659	81
PPT-BIA	p7i2xU-*DA*1	0.01316	227
BIA	p7i2xU-*Lsi*1	0.02841	104
PPT	p7i2xU-*Lsi*1	0.01786	167
PPT-BIA	p7i2xU-*Lsi*1	0.01427	209
BIA	p7i2xU-*PLD*-alpha	0.05556	53
PPT	p7i2xU-*PLD*-alpha	0.01282	233
PPT-BIA	p7i2xU-*PLD*-alpha	0.00714	418
BIA	p7i2xU-*Rubisco*1	0.01786	167
PPT	p7i2xU-*Rubisco*1	0.02139	139
PPT-BIA	p7i2xU-*Rubisco*1	0.01205	248
BIA	p7i2xU-*Rubisco*2	0.04545	65
PPT	p7i2xU-*Rubisco*2	0.00778	384
PPT	p7i2xU-TOR	0.04712	63
PPT-BIA	p7i2xU-TOR	0.01613	185

^a^Treatment means were compared using 95 % confidence intervals

### Molecular analyses of transgenic plants

Overall, we analyzed 273 T0 plants using Southern blot analysis of which 134 transgenic events from ten constructs were confirmed while 73 plants carried only a single copy of the transgene (Table 4). The transformation efficiency (TE) for all constructs used represented 49 % when TE was calculated as the number of T0 plants selected in vitro and transgenic plants identified by Southern blot analyses. Overall, 45 events from nine constructs were selected on PPT alone during the entire selection process and 54 events from ten constructs were obtained on bialaphos alone. In contrast, only 24 events were selected with the mixed selection schema.

**Table 4:** Molecular analyses of ten constructs using Southern blot analysis

Construct	No. of putative transgenic plants	No. of plants analyzed by Southern blot analysis	No. of PCR-positive plants	No. of transgenic events	1 copy	2 copies	3 copies	4 copies	Multi-copy
p7i2x-*At*NCED3	46	33	25	20	9	6	3	2	0
p7i2x-*Ls*i1	63	58	34	29	14	9	6	0	0
p7i2x-*DA*1	20	18	5	11	8	2	1	0	0
p7i2x-*AVP*1	49	38	16	19	11	4	2	1	1
p7i2x-*Rubisco*1	27	26	12	10	5	1	2	2	0
p7i2x-*At*GLU2	7	5	3	2	1	0	1	0	0
p7i2x-*LEW*2	35	32	13	15	8	4	3	0	0
p7i2x-*PLD*-alpha	22	18	6	9	6	2	1	0	0
p7i2x-*Rubisco*2	25	20	7	7	3	1	3	0	0
p7i2x-TOR	27	25	22	12	12	0	0	0	0
Total	321	273	143	134	77	29	22	5	1

### PCR of bar gene

**Fig. 4:**
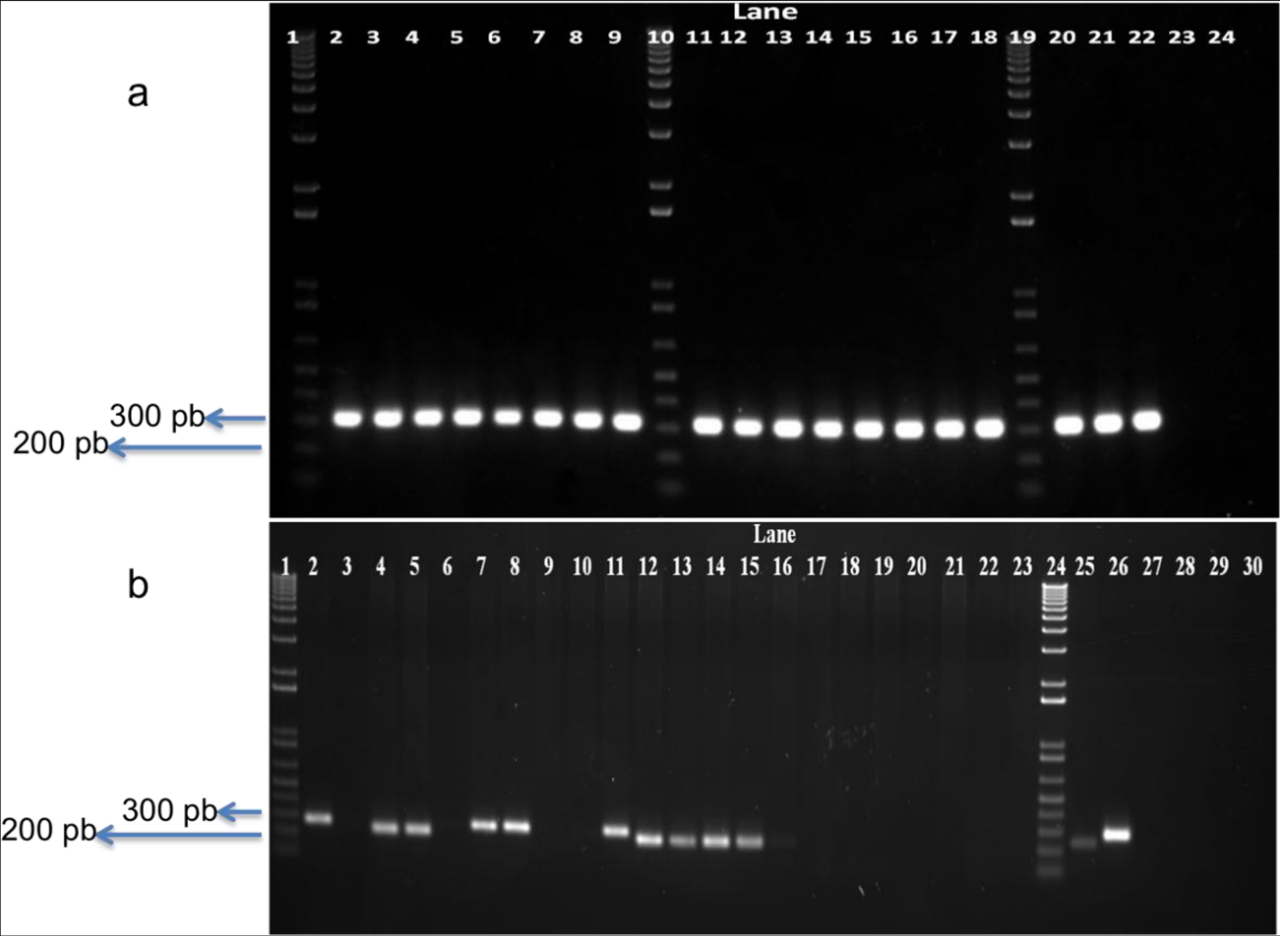
Molecular analysis through conventional PCR. **a** PCR amplification of bar gene. *Lanes 2*–*9*, *11*–*18*: PCR-positive plants. *Lane 20*: plasmid transgenic control. *Lanes 21* and *22*: genomic transgenic controls. *Lane 23*: not transgenic control. *Lane 24*: reaction control. *Lanes 1*, *10*, and *19*: 1-Kb Plus ladder (Invitrogen). Size of band, 318 bp. **b** PCR amplification of backbone integration. *Lanes 1* and *24*: 1-Kb Plus ladder (Invitrogen). *Lanes 2*–*23*: transgenic plants under backbone analysis. *Lane 25*: plasmid p7i2x-*AtNCED3. Lane 26*: plasmid p7i2x-*PLD*-*alpha. Lanes 27*–*29*: not transgenic controls. *Lane 30*: reaction control. Size of bands: 279 bp in constructs p7i2x-*PLD*-*alpha* and p7i2x-*R*u*bisco*1 and 242 bp in remaining plasmids. Note the size difference between plants transformed either with constructs p7i2x-*PLD*-*alpha* and p7i2x-*Rubisco*2 (*Lanes 2*, *7*, *8*, and *11*) or remaining plasmids (*Lanes 4*, *5*, *12*–*15*)

From 321 Curinga rice plants regenerated and analyzed by PCR, 143 showed amplification of the bar gene (Table 4 and Fig. 4a) and were considered as transgenic or PCR positive. Amplification of a band of 318 bp confirmed the transgenic status of plants. This first screening through PCR allowed plant selection for subsequent molecular characterization through Southern blot.

### Southern blot analyses

**Fig. 5:**
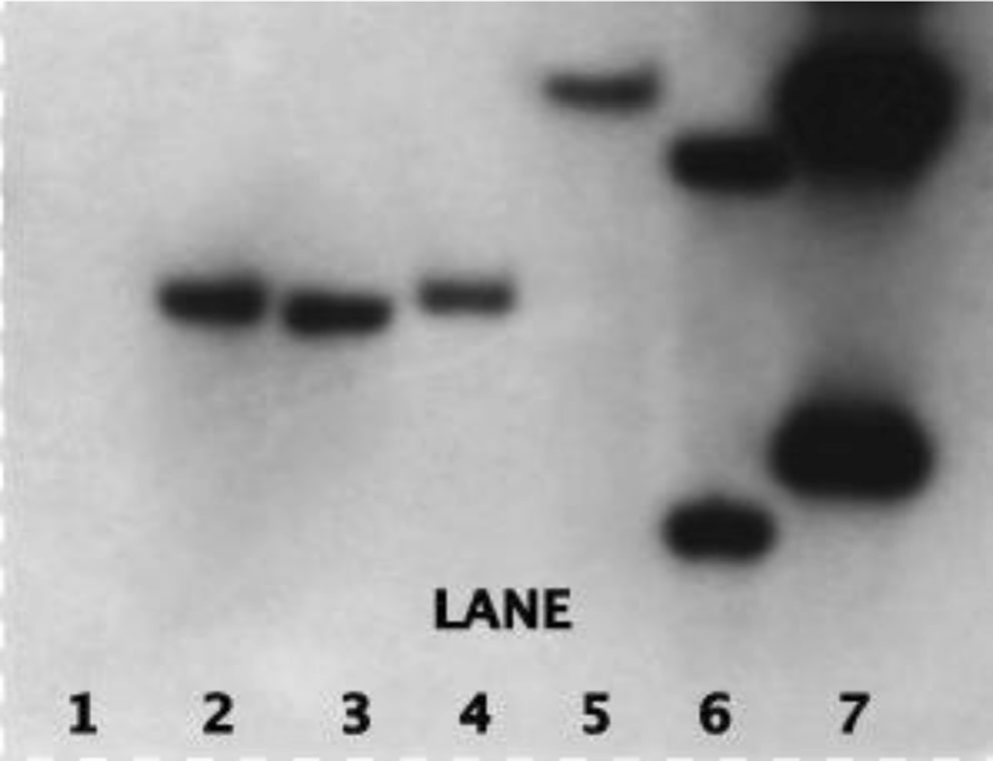
Southern blot detecting bar gene copy number in transgenic rice plants. *Lane 1*: not transgenic control. *Lanes 2–5*: single-copy events. *Lane 6*: two-copy event. *Lane 7*: genomic DNA. *Lanes 2* and *4*, the same transformation event

Through Southern blot analysis, 134 different transgenic events have been identified. Copies of the bar gene integrated in rice genome ranged from one to several (Table 4 and Fig. 5). The maximum copy number of transgene integrated in rice plants were five. Of the total events, 77 had one copy of bar gene which represents 57.46 %, and 29 events had two copies which represents 21.64 %. Our results showed that in total 79.1 % of all transgenic events obtained harbored low copy number of the transgene. Only 16.41 % of events harbored three copies, and 4.47 % events were considered as multi-copy events (≥4 bar gene copies).

### Backbone regions linked to T-DNA

Five pairs of primers covering regions outside the T-DNA borders were used in different PCRs to determine if there was integration of backbone sequences. One hundred and one transgenic plants belonging to all constructs and representing unique events were analyzed by PCR (Table 5 and Fig. 4b). For each border, three backbone regions were amplified demonstrating varying distances from each border. As shown in Table 5, in the case of the right border (RB), 25.7 % (26) of the total transgenic plants analyzed showed PCR amplification of backbone sequences linked to the RB. In the case of the left border (LB), 35.6 % (36) plants showed amplification of backbone regions linked to the LB. In summary, 40.6 % (41) plants had backbone insertions linked to T-DNA (either to RB or LB). Of those 41, less than half had backbone integration from both borders (48.7 %). From all lines that were analyzed, a total of 25 lines (24.75 %) contained insertion toward right border and 36 lines (35.64 %) contained backbone insertion toward left border (data not shown).

**Table 5:** PCR analyses of 101 transgenic plants

No. of transgenic events with only backbone integrated outside RB	No. of transgenic events with only backbone integrated outside LB	No. of transgenic events with integrated backbone outside RB and LB	No. of transgenic events with no backbone sequence integrated
6	15	20	60

### Chlorophenol red test

The color of the medium in this assay changed from purple to yellow indicating that the bar gene activity in transformed rice cells acidified the medium by metabolizing bialaphos. We tested leaf pieces from 45 putative transgenic shoots and untransformed control plants for bar gene expression using the chlorophenol red test (CRT; data not shown). In six cases, the putative transgenic shoots were false negative as determined by the Southern blot analysis, which indicated they were transgenic, carrying the single copy of the transgene. Another plant was identified as negative by CRT; however, it was later identified by Southern blot as transgenic with three copies of the transgene. In contrary, two plants scored positive by CRT but were negative by Southern blot. The results obtained from the CRT and Southern blot showed a 13 % mismatch.

### Leaf brush test with PPT

To evaluate the herbicide resistance level, part of the leaves of transgenic plants and untransformed controls were painted with an aqueous solution of PPT supplemented with Tween-Os20. Overall, 23 transgenic plants from eight constructs (p7i2x-*AVP1*, p7i2x-*Lsi1*, p7i2x-*DA1*, p7i2x-*Rubisco1*, p7i2x-*Rubisco2*, p7i2x-*TOR*, p7i2x-*AtNCED*3, and p7i2x-*GLU*2) showed complete tolerance, i.e., treated leaves stayed green without visible damage, regardless of the transgene copy numbers integrated in the plant genome. However, the leaves of untransformed plants were completely necrotic (data not shown).

### T0 plants and T1 seeds

All transgenic events (107 plants) with low copy numbers integrated in rice genome determined by Southern blot analyses were transferred to the screen house (data not shown). Flowering panicles were covered with the pollination bag to prevent cross pollination, and T0 plants were harvested, additionally dried, and manually trashed. T1 seeds from individual plant were cleaned and stored at 10 °C for the following phenotypic experiments.

## Discussion

The bar gene isolated from *Streptomyces hygroscopicus* encoding the phosphinotricine acetyltransferase (PAT) enzyme which allows resistance to herbicides containing PPT (Thompson et al. 1987, Schomburg and Schomburg 2009) can be used for selection of transgenic plants as initially demonstrated by De Block et al. (1989) for tobacco, potato, and tomato plants. Bialaphos (Ogawa et al. 1973) is a tripeptide antibiotic consisting of PPT, an analog of l-glutamic acid and two l-alanine residues and is produced by fermentation of *Streptomyces hydroscopicus* (Bayer et al. 1972) by Herbiace®, Meiji Seika Ltd. When these residues are removed by peptidases, PPT is able to inhibit glutamine synthetase (GS), the enzyme central to assimilation of ammonium and regulation of nitrogen metabolism (Miflin and Lea 1977). If the GS is inhibited by PPT, accumulation of ammonia results in death of the plant cell (Tachibana et al. 1986). PPT is chemically synthesized by Basta®, Hoechst AG, Germany 1975 (currently Sanofi-Aventis, 2004).

The first herbicide-resistant transgenic rice plants with the bar gene were produced by electroporation (Dekeyser et al. 1989; Toki et al. 1992) by polyethylene glycol (PEG)-mediated transformation of rice protoplast (Datta et al. 1990) or by cell suspension cultures transformed via particle bombardment (Cao et al. 1992; Xu et al. 1996).

*Agrobacterium*-mediated transformation using the bar gene in combination with anti-necrotic treatments was reported for *japonica* rice R-321 by Enrıquez-Obregon et al. (1999). The following authors used GFP and bar selection with different *japonica* rice genotypes (Jang et al. 1999; Nakamura et al. 2010) or in combination with transformation histone enhancer (Zheng et al. 2009). Recently, Duan et al. (2012) reported a high-throughput protocol for *japonica* cv. Nipponbare and Wanjing 97 with mannose and bar selection. It is difficult to compare our results with those previously published due to the different rice genotypes used as well as promoters driving the bar gene. Furthermore, we observed big differences among our experiments when we compared the number of calli needed for one transgenic plant production if comparing the ten constructs with which we worked.

In our study, the majority (73.1 %) of the transgenic events produced using *Agrobacterium*-mediated transformation at most two copies of the transgene. This confirms that transformation mediated by *Agrobacterium* favors the regeneration of low copy transgenic plants. Previous studies support this finding, as demonstrated in maize, where Shou et al. (2004) obtained regeneration of 92 % of low copy number events (less than three copies) and sorghum where Zhou et al. (2000) reported 72 % of single-copy events by using *Agrobacterium*. Dai et al. (2001) also reported the regeneration of low copy events in rice transgenic plants. These reports confirm the central role of *Agrobacterium* in efficient transformation protocols for agronomically important cereal crops such as rice, wheat, maize, barley, and sorghum (Cheng et al. 2006).

A large portion (40.6 %) of rice transgenic plants obtained in our study had backbone sequences linked to T-DNA. Previous studies identified vector backbone sequences linked to T-DNA in a range of species transformed by *Agrobacterium* including barley (Lange et al. 2006), Arabidopsis (De Buck et al. 2000), tobacco (Kononov et al. 1997 and De Buck et al. 2000), wheat (Wu et al. 2006) and rice (Kim et al. 2003; Afolabi et al. 2004; Kuraya et al. 2004 and Yin and Wang 2000). These reports also show significant ratios of backbone insertions ranking from 20 % (De Buck et al. 2000) to 75 % (Kononov et al. 1997) in transgenic plants not including rice and from 33 % (Yin and Wang 2000) to 92 % (Kuraya et al. 2004) in rice transgenic plants. One explanation for the integration of vector backbone appears to be inefficient recognition of the left and right border as initiation and termination sites for T-DNA transfer resulting in read-through at both borders (De Buck et al. 2000). Fang et al. (2000), in study done on rice, identified two types of vector backbone sequence: directly linked to the T-DNA across either LB or RB (type I) and backbone sequence not directly linked to a T-DNA border (type II). In our study, we focused only on type I backbone sequences integration. We found 40.6 % of this type of insertion, and it is only slightly higher compared to what has previously been reported (37.5 %). Moreover, as it was shoved by Zuniga, 2014, CIAT (personal communication), using the chromosome walking approach, the adjacent region of the T-DNA integration in rice can have different configuration closed to RB.

The original chlorophenol red assay described by Kramer et al. (1993) for maize callus selected with PPT was modified for transgenic plants selected with D-mannose for maize and wheat (Wright et al. 2001), for *japonica* rice (Lucca et al. 2001), for chickpea (Patil et al. 2009), and for transgenic cowpea plants (Bakshi et al. 2012). All of these authors support the high correlation between the CRT and PCR results. Based on our Southern blot results compared to the CRT, we found discrepancies in 13 % of the T0 plants tested. The bar gene expression in T0 rice plants together with the herbicide resistance of transgenic plants can be tested by the simple leaf painting test, using PPT, bialaphos, or the herbicides Basta (Rathore et al. 1993) and Challenge (Wu et al. 2003). As we found, the results from this assay were highly correlated with the PCR results (Rasco-Gaunt et al. 1999) where all transgenic plants tested positive by PCR with copy number identified by Southern blot analyses. Positive correlation between molecular analyses and bar gene expression resulting herbicide resistance was also described in other crops, for example wheat (Wu et al. 2006), carrot (Jayaraj et al. 2008), or cassava (Koehorst-van Putten et al. 2012).

By producing over hundreds of transgenic events carrying different genes of interest, we created a good basis for the further phenotyping experiments to evaluate the performance for agronomically important traits, as well as we have developed a transformation protocol for an important commercial Brazilian upland rice cultivar with herbicide resistance.
